# *trans*-Bis(perchlorato-κ*O*)tetra­kis(1*H*-pyrazole-κ*N* ^2^)copper(II)

**DOI:** 10.1107/S1600536808030080

**Published:** 2008-09-24

**Authors:** Frank T. Edelmann, Dieter E. Kaufmann, Steffen Blaurock, Thomas Wagner, Viktor Zapol’skii

**Affiliations:** a Chemisches Institut, Otto-von-Guericke-Universität Magdeburg, Universitätsplatz 2, D-39106 Magdeburg, Germany; b Institut für Organische Chemie, Technische Universität Clausthal, Leibnizstrasse 6, D-38678 Clausthal-Zellerfeld, Germany

## Abstract

The title compound, [Cu(ClO_4_)_2_(C_3_H_4_N_2_)_4_], was obtained unexpectedly by the reaction of copper(II) perchlorate hexa­hydrate with equimolar amounts of 1-chloro-1-nitro-2,2,2-tripyrazolylethane in methanol solution. The crystal structure comprises octa­hedrally coordinated Cu^2+^ ions, located on an inversion centre, with four pyrazole ligands in the equatorial plane. The average Cu—N distance is 2.000 (1) Å. Two perchlorate ions are coordinated to copper in *trans* positions [Cu—O = 2.4163 (11) Å].

## Related literature

For general background on weakly coordinating anions, see: Gowda *et al.* (1984 ▶); Rosenthal (1973 ▶); Strauss (1993 ▶). For previous literature on the title compound, see: Misra *et al.* (1998 ▶); Reedijk (1969 ▶);  Sastry *et al.* (1986 ▶). For 1-chloro-1-nitro-2,2,2-tripyrazolylethane, see: Zapol’skii & Kaufmann (2008 ▶). 
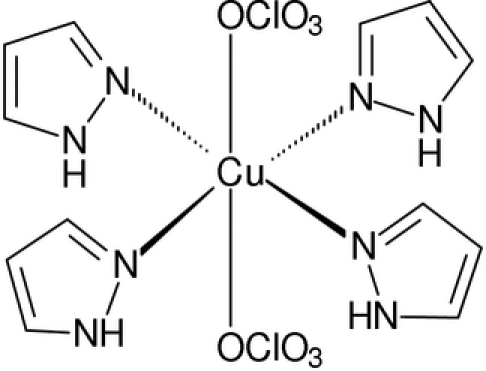


## Experimental

### 

#### Crystal data

[Cu(ClO_4_)_2_(C_3_H_4_N_2_)_4_]
*M* *_r_* = 534.77
Monoclinic, 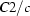

*a* = 14.1537 (11) Å
*b* = 9.9483 (5) Å
*c* = 15.7414 (12) Å
β = 114.946 (6)°
*V* = 2009.7 (3) Å^3^
*Z* = 4
Mo *K*α radiation
μ = 1.41 mm^−1^
*T* = 173 (2) K
0.40 × 0.30 × 0.20 mm

#### Data collection

Stoe IPDS 2T diffractometer
Absorption correction: integration (*X-RED*; Stoe & Cie, 2001 ▶) *T*_min_ = 0.606, *T*_max_ = 0.881
9046 measured reflections
2687 independent reflections
2518 reflections with *I* > 2σ(*I*)
*R*_int_ = 0.017

#### Refinement

*R*[*F* ^2^ > 2σ(*F* ^2^)] = 0.025
*wR*(*F* ^2^) = 0.068
*S* = 0.99
2687 reflections
174 parameters
All H-atom parameters refined
Δρ_max_ = 0.37 e Å^−3^
Δρ_min_ = −0.34 e Å^−3^

### 

Data collection: *X-AREA* (Stoe & Cie, 2001 ▶); cell refinement: *X-AREA*; data reduction: *X-RED* (Stoe & Cie, 2001 ▶); program(s) used to solve structure: *SHELXS97* (Sheldrick, 2008 ▶); program(s) used to refine structure: *SHELXL97* (Sheldrick, 2008 ▶); molecular graphics: *SHELXTL-Plus* (Sheldrick, 2008 ▶); software used to prepare material for publication: *SHELXTL-Plus*.

## Supplementary Material

Crystal structure: contains datablocks I, global. DOI: 10.1107/S1600536808030080/bt2785sup1.cif

Structure factors: contains datablocks I. DOI: 10.1107/S1600536808030080/bt2785Isup2.hkl

Additional supplementary materials:  crystallographic information; 3D view; checkCIF report

## Figures and Tables

**Table 1:** Hydrogen-bond geometry (Å, °)

*D*—H⋯*A*	*D*—H	H⋯*A*	*D*⋯*A*	*D*—H⋯*A*
N2—H2*N*⋯O3^i^	0.81 (3)	2.25 (3)	3.0517 (19)	171 (2)
N2—H2*N*⋯O4^i^	0.81 (3)	2.58 (2)	3.0722 (17)	121 (2)
N4—H4*N*⋯O4^i^	0.79 (2)	2.24 (2)	2.8124 (17)	129.3 (17)
N4—H4*N*⋯O2^ii^	0.79 (2)	2.50 (2)	3.1580 (17)	141.5 (17)

Symmetry codes: (i) 
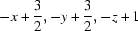
; (ii) 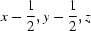.

**Fig. 1.**
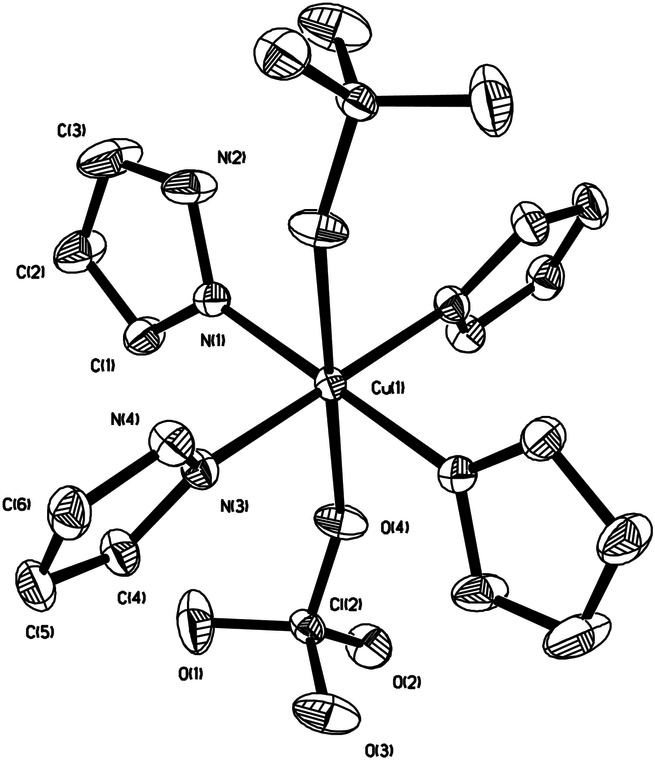
The molecule of the title compound in the crystal. Thermal ellipsoids represent 50% probability levels. H-Atom radii are arbitrary.

## supplementary crystallographic information

### Comment

Today it is generally recognized that the classical "noncoordinating" anions such as ClO_4_^-^, BF_4_^-^ or PF_6_^-^ can coordinate to metal ions from all regions of the periodic table (Strauss, 1993; Rosenthal, 1973; Gowda *et al.* , 1984). In the course of an investigation on the coordination chemistry of various azolyl-nitrochloroalkanes (Zapol'skii & Kaufmann, 2008), we studied the reaction of copper(II) perchlorate hexahydrate with equimolar amounts of 1-chloro-1-nitro-2,2,2-tris(pyrazolyl)ethane, Cl(NO_2_)CH—C(C_3_H_3_N_2_)_3_, in methanol solution. Quite unexpectedly, complete degradation of the starting material took place during the course of this reaction. Direct crystallization from the concentrated reaction mixture afforded dark blue single-crystals. An X-ray structure determination revealed the presence of the title compound, *trans*-bis(perchlorato)-tetrakis(pyrazole)copper(II). The formation of free pyrazol can only be explained by a solvolytic degradation of the starting material. This degradation must take place on a large extent as the isolated yield of the title compound was 64%. The complex *trans*-bis(perchlorato)-tetrakis(pyrazole)copper(II) has been mentioned three times before in the earlier literature, but structural characterization was lacking until now.  Reedijk (1969) first decribed the preparation of the title compound by direct treatment of copper perchlorate with pyrazole. The compound was characterized and identified by elemental analysis and physical measurements. Infrared spectroscopy evidently showed coordination of the perchlorate anions to the central copper(2+) ion. This was now confirmed by the present X-ray diffraction study. The structure of the title compound is shown below. Dimensions are available in the archived CIF. In the solid state, the title compound comprises octahedral molecules in which the central Cu^2+^ ion is surrounded by four neutral pyrazole ligand in the equatorial plane. The average Cu—N distance is 2.000 (1) Å. Two perchlorate ions are coordinated to copper in the *trans* positions (Cu—O 2.4163 (11) Å).

### Experimental

The title compound was obtained as an unexpected product from a reaction of copper(II) perchlorate hexahydrate with equimolar amounts of 1-chloro-1-nitro-2,2,2-tris(pyrazolyl)ethane, Cl(NO_2_)CH—C(C_3_H_3_N_2_)_3_, in methanol solution. Solid 1-chloro-1-nitro-2,2,2-tris(pyrazolyl)ethane (0.56 g) was added to a solution of Cu(ClO_4_)_2 _*x* 6H_2_O in methanol (50 ml) and the mixture was stirred at ambient temperature for 2 h. The solution was concentrated under vacuum to a total volume of *ca* 15 ml and allowed to stand undisturbed at room temperature. After several days, dark blue single-crystals of the title compound were obtained in 64% yield (0.45 g, based on Cu(ClO_4_)_2 _*x* 6H_2_O). Anal. calcd for C_12_H_16_Cl_2_CuN_8_O_8_ (534.75 g mol^-1^): C 26.85, H 3.04; found: C 26.11, H 3.18%.

### Refinement

All H atoms were freely refined.

### Crystal data

**Table:**

[Cu(ClO_4_)_2_(C_3_H_4_N_2_)_4_]	*F*(000) = 1084
*M**_r_* = 534.77	*D*_x_ = 1.767 Mg m^−^^3^
Monoclinic, *C*2/*c*	Mo *K*α radiation, λ = 0.71073 Å
Hall symbol: -C2yc	Cell parameters from 16684 reflections
*a* = 14.1537 (11) Å	θ = 2.6–29.5°
*b* = 9.9483 (5) Å	µ = 1.41 mm^−^^1^
*c* = 15.7414 (12) Å	*T* = 173 K
β = 114.946 (6)°	Prism, green
*V* = 2009.7 (3) Å^3^	0.40 × 0.30 × 0.20 mm
*Z* = 4	

### Data collection

**Table:**

Stoe IPDS 2T diffractometer	2687 independent reflections
Radiation source: sealed X-ray tube, 12 x 0.4 mm long-fine focus	2518 reflections with *I* > 2σ(*I*)
plane graphite	*R*_int_ = 0.017
Detector resolution: 6.67 pixels mm^-1^	θ_max_ = 29.2°, θ_min_ = 2.6°
rotation method scans	*h* = −19→19
Absorption correction: integration (XRED; Stoe & Cie, 2001)	*k* = −13→13
*T*_min_ = 0.606, *T*_max_ = 0.881	*l* = −21→21
9046 measured reflections	

### Refinement

**Table:**

Refinement on *F*^2^	Primary atom site location: structure-invariant direct methods
Least-squares matrix: full	Secondary atom site location: difference Fourier map
*R*[*F*^2^ > 2σ(*F*^2^)] = 0.025	Hydrogen site location: inferred from neighbouring sites
*wR*(*F*^2^) = 0.068	All H-atom parameters refined
*S* = 0.99	*w* = 1/[σ^2^(*F*_o_^2^) + (0.0449*P*)^2^ + 1.7635*P*] where *P* = (*F*_o_^2^ + 2*F*_c_^2^)/3
2687 reflections	(Δ/σ)_max_ = 0.001
174 parameters	Δρ_max_ = 0.37 e Å^−^^3^
0 restraints	Δρ_min_ = −0.34 e Å^−^^3^

### Special details

**Table:**

Geometry. All e.s.d.'s (except the e.s.d. in the dihedral angle between two l.s. planes) are estimated using the full covariance matrix. The cell e.s.d.'s are taken into account individually in the estimation of e.s.d.'s in distances, angles and torsion angles; correlations between e.s.d.'s in cell parameters are only used when they are defined by crystal symmetry. An approximate (isotropic) treatment of cell e.s.d.'s is used for estimating e.s.d.'s involving l.s. planes.
Refinement. Refinement of *F*^2^ against ALL reflections. The weighted *R*-factor *wR* and goodness of fit *S* are based on *F*^2^, conventional *R*-factors *R* are based on *F*, with *F* set to zero for negative *F*^2^. The threshold expression of *F*^2^ > σ(*F*^2^) is used only for calculating *R*-factors(gt) *etc*. and is not relevant to the choice of reflections for refinement. *R*-factors based on *F*^2^ are statistically about twice as large as those based on *F*, and *R*- factors based on ALL data will be even larger.

### Fractional atomic coordinates and isotropic or equivalent isotropic displacement parameters (Å^2^)

**Table:**

	*x*	*y*	*z*	*U*_iso_*/*U*_eq_	
Cu1	0.7500	0.7500	0.5000	0.01532 (8)	
Cl2	0.90323 (2)	1.06663 (3)	0.56166 (2)	0.01900 (9)	
O1	0.98696 (11)	1.03113 (16)	0.64859 (9)	0.0454 (3)	
O2	0.94232 (9)	1.14470 (12)	0.50756 (8)	0.0312 (2)	
O3	0.82438 (10)	1.13865 (14)	0.57726 (10)	0.0412 (3)	
O4	0.85586 (8)	0.94534 (11)	0.51056 (8)	0.0283 (2)	
N1	0.87702 (9)	0.63692 (11)	0.55955 (8)	0.0191 (2)	
N2	0.87750 (10)	0.50290 (13)	0.54924 (11)	0.0299 (3)	
N3	0.74610 (9)	0.77577 (12)	0.62524 (8)	0.0186 (2)	
N4	0.67422 (9)	0.71389 (13)	0.64642 (9)	0.0218 (2)	
C1	0.97459 (11)	0.66995 (15)	0.61591 (11)	0.0254 (3)	
C2	1.03738 (12)	0.55634 (17)	0.64268 (14)	0.0364 (4)	
C3	0.97222 (14)	0.45238 (18)	0.59796 (17)	0.0435 (5)	
C4	0.80535 (11)	0.84451 (16)	0.70182 (10)	0.0241 (3)	
C5	0.77179 (12)	0.82591 (17)	0.77210 (10)	0.0274 (3)	
C6	0.68758 (13)	0.74111 (16)	0.73397 (11)	0.0262 (3)	
H2N	0.825 (2)	0.460 (3)	0.5206 (17)	0.045 (6)*	
H4N	0.6330 (15)	0.666 (2)	0.6088 (14)	0.023 (4)*	
H1	0.9926 (19)	0.764 (2)	0.6332 (17)	0.033 (5)*	
H2	1.107 (2)	0.554 (2)	0.6810 (17)	0.047 (6)*	
H3	0.984 (2)	0.356 (3)	0.599 (2)	0.063 (8)*	
H4	0.8596 (16)	0.894 (2)	0.7022 (15)	0.032 (5)*	
H5	0.7974 (19)	0.866 (3)	0.8298 (18)	0.048 (6)*	
H6	0.6457 (18)	0.705 (2)	0.7603 (17)	0.042 (6)*	

### Atomic displacement parameters (Å^2^)

**Table:**

	*U*^11^	*U*^22^	*U*^33^	*U*^12^	*U*^13^	*U*^23^
Cu1	0.01307 (11)	0.01805 (12)	0.01317 (11)	−0.00047 (7)	0.00391 (8)	−0.00027 (7)
Cl2	0.02026 (15)	0.01988 (15)	0.01697 (14)	−0.00576 (10)	0.00797 (11)	−0.00313 (10)
O1	0.0377 (7)	0.0568 (8)	0.0245 (6)	−0.0117 (6)	−0.0037 (5)	0.0078 (6)
O2	0.0409 (6)	0.0281 (5)	0.0297 (6)	−0.0113 (5)	0.0197 (5)	0.0010 (4)
O3	0.0382 (6)	0.0409 (7)	0.0548 (8)	−0.0038 (5)	0.0296 (6)	−0.0205 (6)
O4	0.0268 (5)	0.0224 (5)	0.0380 (6)	−0.0107 (4)	0.0158 (5)	−0.0110 (4)
N1	0.0174 (5)	0.0182 (5)	0.0195 (5)	0.0002 (4)	0.0057 (4)	−0.0006 (4)
N2	0.0197 (6)	0.0200 (6)	0.0445 (8)	−0.0023 (5)	0.0080 (5)	−0.0083 (5)
N3	0.0173 (5)	0.0216 (5)	0.0167 (5)	−0.0016 (4)	0.0070 (4)	−0.0004 (4)
N4	0.0205 (5)	0.0250 (5)	0.0215 (6)	−0.0042 (4)	0.0104 (5)	−0.0016 (5)
C1	0.0183 (6)	0.0208 (6)	0.0296 (7)	−0.0009 (5)	0.0028 (5)	0.0002 (5)
C2	0.0189 (7)	0.0262 (7)	0.0507 (10)	0.0035 (6)	0.0015 (6)	0.0011 (7)
C3	0.0273 (8)	0.0215 (7)	0.0708 (13)	0.0049 (6)	0.0098 (8)	−0.0038 (8)
C4	0.0218 (6)	0.0291 (7)	0.0180 (6)	−0.0042 (5)	0.0051 (5)	−0.0017 (5)
C5	0.0278 (7)	0.0359 (8)	0.0156 (6)	0.0015 (6)	0.0063 (5)	−0.0018 (5)
C6	0.0272 (7)	0.0327 (8)	0.0216 (6)	0.0031 (5)	0.0131 (6)	0.0044 (5)

### Geometric parameters (Å, °)

**Table:**

Cu1—N1^i^	1.9887 (11)	N3—C4	1.3310 (18)
Cu1—N1	1.9887 (11)	N3—N4	1.3460 (16)
Cu1—N3^i^	2.0117 (12)	N4—C6	1.3373 (19)
Cu1—N3	2.0117 (12)	N4—H4N	0.79 (2)
Cu1—O4^i^	2.4164 (10)	C1—C2	1.389 (2)
Cu1—O4	2.4164 (10)	C1—H1	0.98 (2)
Cl2—O1	1.4261 (12)	C2—C3	1.367 (2)
Cl2—O2	1.4255 (11)	C2—H2	0.91 (3)
Cl2—O3	1.4318 (12)	C3—H3	0.97 (3)
Cl2—O4	1.4483 (10)	C4—C5	1.388 (2)
N1—C1	1.3299 (17)	C4—H4	0.91 (2)
N1—N2	1.3435 (17)	C5—C6	1.375 (2)
N2—C3	1.331 (2)	C5—H5	0.91 (3)
N2—H2N	0.81 (3)	C6—H6	0.93 (2)
			
N1^i^—Cu1—N1	180.000 (1)	C3—N2—H2N	125.5 (18)
N1^i^—Cu1—N3^i^	90.18 (5)	N1—N2—H2N	123.2 (18)
N1—Cu1—N3^i^	89.82 (5)	C4—N3—N4	105.30 (11)
N1^i^—Cu1—N3	89.82 (5)	C4—N3—Cu1	133.31 (10)
N1—Cu1—N3	90.18 (5)	N4—N3—Cu1	121.37 (9)
N3^i^—Cu1—N3	180.0	C6—N4—N3	111.69 (12)
N1^i^—Cu1—O4^i^	90.65 (4)	C6—N4—H4N	129.1 (14)
N1—Cu1—O4^i^	89.35 (4)	N3—N4—H4N	119.1 (14)
N3^i^—Cu1—O4^i^	95.89 (4)	N1—C1—C2	110.67 (13)
N3—Cu1—O4^i^	84.11 (4)	N1—C1—H1	119.8 (14)
N1^i^—Cu1—O4	89.35 (4)	C2—C1—H1	129.5 (14)
N1—Cu1—O4	90.65 (4)	C3—C2—C1	104.66 (14)
N3^i^—Cu1—O4	84.11 (4)	C3—C2—H2	128.5 (16)
N3—Cu1—O4	95.89 (4)	C1—C2—H2	126.8 (16)
O4^i^—Cu1—O4	180.00 (6)	N2—C3—C2	107.94 (15)
O1—Cl2—O2	109.46 (8)	N2—C3—H3	120.5 (16)
O1—Cl2—O3	110.60 (9)	C2—C3—H3	131.5 (16)
O2—Cl2—O3	110.90 (8)	N3—C4—C5	110.90 (13)
O1—Cl2—O4	109.23 (8)	N3—C4—H4	119.4 (13)
O2—Cl2—O4	109.12 (7)	C5—C4—H4	129.6 (13)
O3—Cl2—O4	107.49 (7)	C6—C5—C4	105.01 (13)
Cl2—O4—Cu1	146.78 (7)	C6—C5—H5	127.3 (15)
C1—N1—N2	105.61 (11)	C4—C5—H5	127.6 (15)
C1—N1—Cu1	130.74 (10)	N4—C6—C5	107.09 (13)
N2—N1—Cu1	123.64 (9)	N4—C6—H6	122.9 (15)
C3—N2—N1	111.12 (13)	C5—C6—H6	130.0 (15)
			
O1—Cl2—O4—Cu1	71.01 (15)	N1—Cu1—N3—C4	70.96 (14)
O2—Cl2—O4—Cu1	−169.36 (12)	N3^i^—Cu1—N3—C4	0(100)
O3—Cl2—O4—Cu1	−49.03 (15)	O4^i^—Cu1—N3—C4	160.28 (14)
N1^i^—Cu1—O4—Cl2	81.05 (13)	O4—Cu1—N3—C4	−19.72 (14)
N1—Cu1—O4—Cl2	−98.95 (13)	N1^i^—Cu1—N3—N4	72.95 (11)
N3^i^—Cu1—O4—Cl2	171.30 (14)	N1—Cu1—N3—N4	−107.05 (11)
N3—Cu1—O4—Cl2	−8.70 (14)	N3^i^—Cu1—N3—N4	0(100)
O4^i^—Cu1—O4—Cl2	111 (100)	O4^i^—Cu1—N3—N4	−17.73 (10)
N1^i^—Cu1—N1—C1	−140 (100)	O4—Cu1—N3—N4	162.27 (10)
N3^i^—Cu1—N1—C1	108.25 (14)	C4—N3—N4—C6	−0.55 (16)
N3—Cu1—N1—C1	−71.75 (14)	Cu1—N3—N4—C6	177.94 (10)
O4^i^—Cu1—N1—C1	−155.85 (14)	N2—N1—C1—C2	−0.78 (19)
O4—Cu1—N1—C1	24.15 (14)	Cu1—N1—C1—C2	178.35 (12)
N1^i^—Cu1—N1—N2	39 (100)	N1—C1—C2—C3	0.9 (2)
N3^i^—Cu1—N1—N2	−72.76 (12)	N1—N2—C3—C2	0.2 (2)
N3—Cu1—N1—N2	107.24 (12)	C1—C2—C3—N2	−0.6 (2)
O4^i^—Cu1—N1—N2	23.13 (12)	N4—N3—C4—C5	0.38 (17)
O4—Cu1—N1—N2	−156.87 (12)	Cu1—N3—C4—C5	−177.85 (11)
C1—N1—N2—C3	0.4 (2)	N3—C4—C5—C6	−0.09 (18)
Cu1—N1—N2—C3	−178.83 (14)	N3—N4—C6—C5	0.51 (17)
N1^i^—Cu1—N3—C4	−109.04 (14)	C4—C5—C6—N4	−0.24 (17)

Symmetry codes: (i) −*x*+3/2, −*y*+3/2, −*z*+1.

### Hydrogen-bond geometry (Å, °)

**Table:**

*D*—H···*A*	*D*—H	H···*A*	*D*···*A*	*D*—H···*A*
N2—H2N···O3^i^	0.81 (3)	2.25 (3)	3.0517 (19)	171 (2)
N2—H2N···O4^i^	0.81 (3)	2.58 (2)	3.0722 (17)	121 (2)
N4—H4N···O4^i^	0.79 (2)	2.24 (2)	2.8124 (17)	129.3 (17)
N4—H4N···O2^ii^	0.79 (2)	2.50 (2)	3.1580 (17)	141.5 (17)

Symmetry codes: (i) −*x*+3/2, −*y*+3/2, −*z*+1; (ii) *x*−1/2, *y*−1/2, *z*.
